# Actin Disorganization Plays a Vital Role in Impaired Embryonic Development of *In Vitro*-Produced Mouse Preimplantation Embryos

**DOI:** 10.1371/journal.pone.0130382

**Published:** 2015-06-15

**Authors:** Kun Tan, Lei An, Shu-Min Wang, Xiao-Dong Wang, Zhen-Ni Zhang, Kai Miao, Lin-Lin Sui, Shu-Zhi He, Jing-Zhou Nie, Zhong-Hong Wu, Jian-Hui Tian

**Affiliations:** Key Laboratory of Animal Genetics, Breeding and Reproduction of the Ministry of Agriculture, National Engineering Laboratory for Animal Breeding, College of Animal Science and Technology, China Agricultural University, Beijing, P. R. China; Michigan State University, UNITED STATES

## Abstract

Assisted reproductive technology (ART) is being increasingly applied to overcome infertility. However, the *in vitro* production process, the main procedure of ART, can lead to aberrant embryonic development and health-related problems in offspring. Understanding the mechanisms underlying the ART-induced side effects is important to improve the ART process. In this study, we carried out comparative transcriptome profiling between *in vivo*- (IVO) and *in vitro*- produced (IVP) mouse blastocysts. Our results suggested that aberrant actin organization might be a major factor contributing to the impaired development of IVP embryos. To test this, we examined the effect of actin disorganization on the development of IVP preimplantation embryos. Specific disruption of actin organization by cytochalasin B (CB) indicated that well-organized actin is essential for *in vitro* embryonic development. Supplementing the culture medium with 10^–9^ M melatonin, a cytoskeletal modulator in adult somatic cells, significantly reversed the disrupted expression patterns of genes related to actin organization, including *Arhgef2*, *Bcl2*, *Coro2b*, *Flnc*, and *Palld*. Immunofluorescence analysis showed that melatonin treatment of IVP embryos significantly improved the distribution and organization of actin filaments (F-actin) from the 8-cell stage onwards. More importantly, we found that melatonin alleviated the CB-mediated aberrant F-actin distribution and organization and rescued CB-induced impaired embryonic development. This is the first study to indicate that actin disorganization is implicated in impaired development of IVP embryos during the preimplantation stage. We also demonstrated that improving actin organization is a promising strategy to optimize existing IVP systems.

## Introduction

The number of patients receiving assisted reproductive technology (ART) to treat infertility has increased annually. It is estimated that approximately 1.5 million ART cycles are performed annually worldwide, resulting in 5,000,000 live births [1]. However, ART is associated with risks, such as aberrant embryonic development and health-related problems in the offspring [2]. Studies have shown that *in vitro* fertilization (IVF) and *in vitro* culture (IVC), the main procedures of ART, have a profound effect on gene/protein expression patterns and phenotypes in pre- and post-implantation mouse embryos [3, 4]. Moreover, ART is associated with long-term effects on the offspring’s health, such as fetal complications [5], and an increased risk of diseases in childhood and adulthood [6]. Using high-throughput methods, such as microarrays and RNA sequencing (RNA-seq), many studies have reported that a series of biological processes, including energy metabolism [5], genetic information processing [7], and epigenetic modifications [8, 9], are disrupted in IVP embryos. However, these deductions were generally based on the functional analysis of high-throughput data, which need to be further confirmed. Moreover, in-depth understanding of mechanisms of these effects would be beneficial to optimize the IVP processes.

Thus far, improvements of *in vitro* embryonic development have primarily depended on modifying the *in vitro* culture system. Some improvements have shown beneficial effects on *in vitro* embryonic development, and optimizations have included the use of chemically defined culture media [10], two-step sequential culture system (e.g., G1/G2 [11]), and supplementation of medium with specific physiological factors (e.g., glutathione [12], cysteine [13] and melatonin [14]). Melatonin (*N*-acetyl-5-methoxytryptamine), a natural molecule mainly secreted by the pineal gland in mammals, apparently improves *in vitro* embryonic development in mice [14], cattle [15], sheep [16], pigs [17] and humans [18]. Previously, the pervasive antioxidant and apoptosis inhibition abilities [15, 17, 19] of melatonin were found thought to be responsible for the improvement of *in vitro* embryonic development. However, melatonin has multiple functions. It participates in many cellular functions, including prompting mitochondrial biogenesis [20], regulating nuclear transcriptional activity [21] and facilitating gap-junction commutation [22].

In this study, we carried out comparative transcriptome profiling between *in vivo*- (IVO) and *in vitro*- (IVP) produced mouse blastocysts. Functional clustering showed that aberrant actin organization might be a major factor contributing to the impaired development of IVP preimplantation embryos. The results of CB-induced actin disorganization further suggested that well-organized actin organization is essential for *in vitro* embryonic development. These results also implied that actin organization could be a target for improving *in vitro* embryonic development. In previous studies, melatonin was shown to act as a cytoskeletal modulator in somatic cells and cancer cells [23]. Therefore, we hypothesized that melatonin might also participate in actin organization during *in vitro* embryonic development, which contributes to the beneficial effect it exerts on *in vitro* embryonic development. Our results showed that melatonin could reverse the dysregulated expression of genes associated with actin organization, and alleviated the actin disorganization in IVP preimplantation embryos. Moreover, melatonin could rescue the cytochalasin B (CB)-induced actin disorganization and impaired preimplantation embryonic development. Our study, indicated, for the first time, that actin disorganization is a major factor in the impaired development of IVP embryos during the preimplantation stage. More importantly, we also demonstrated that improving actin organization is a promising strategy to optimize existing IVP systems.

## Materials and Methods

### Ethics Statement

The protocols for the animal studies were approved by, and performed in accordance with, the requirements of the Institutional Animal Care and Use Committee of China Agricultural University.

### Animals

We used female Institute for Cancer Research (ICR) mice aged 7 to 8- weeks and male ICR mice aged 12 to 14 weeks. The mice were fed *ad libitum* and housed under controlled lighting conditions (12:12-h light:dark photoperiod).

### Chemicals

All chemicals used were purchased from the Sigma-Aldrich Chemical Company (St. Louis, MO, USA) unless otherwise specified.

### Embryo Preparation

The female mice were superovulated by intraperitoneal injection of 5 IU pregnant mare serum gonadotropin (PMSG) and 5 IU of human chorionic gonadotropin (hCG) after 47 h. In the IVO group, the superovulated female mice were mated individually with male mice. The following morning, successful mating was confirmed by detecting the presence of a vaginal plug.

In the IVP group, IVF was performed as previously described [4]. Briefly, cumulus-enclosed oocyte complexes (COCs) were collected from the ampullae 14 h after the hCG injection, and the cumulus cells were removed by digesting the COCs with hyaluronidase for 3−5 min. The oocytes were first rinsed in human tubal fluid (HTF) medium (Sage, Bedminster, NJ, USA), and placed in 60-μL drops of HTF medium, covered with paraffin oil and equilibrated overnight at 37°C and 5% CO_2_. Sperm was obtained from the cauda epididymis and capacitated for 1 h in HTF medium at 37°C and 5% CO_2_. Sperm insemination was carried out 15 h after hCG injection. After 4 h in the incubator, the oocytes/embryos were washed several times in potassium simplex optimization medium containing amino acids (KSOM + AA; Millipore, Billerica, MA, USA) and transferred to 60-μL drops of KSOM + AA medium covered with paraffin oil. The embryos were cultured at 37°C with 5% CO_2_.

### Treatment with melatonin or CB

The treatment of embryos with CB was performed as previously described [24], with minor modifications. In brief, CB was dissolved as a stock solution (20 mg/ml) in dimethyl sulfoxide (DMSO) and stored at −20°C. The stock solution was later diluted to the working concentrations (5μg/ml, 10 μg/ml and 20 μg/ml) in KSOM + AA medium, before use. We set two controls: untreated control (IVP group) and vehicle control (supplementation with DMSO; 0.1% v/v, the same amount of DMSO as the embryos receiving 20 μg/ml CB treatment).

Melatonin was dissolved as a stock solution (15mM) in DMSO and stored at −80°C. The stock solution was later diluted to the working concentrations (10^−7^~10^−10^ M) in KSOM + AA medium before use. For melatonin treatment, the concentration of DMSO supplemented into the KSOM + AA medium (< 0.001% v/v, the same amount of DMSO as the embryos receiving melatonin treatment in 10^−7^ M group) was very low compared with the CB-vehicle control (0.1% v/v); therefore, we did not perform this vehicle control for melatonin treatment.

After IVF, zygotes were further cultured in KSOM + AA medium supplemented with melatonin or/ and CB. For CB treatment, the zygotes were treated for 3 h, and then removed from the medium containing CB. After washing at least three times in KSOM + AA medium, embryos were further cultured in CB-free KSOM + AA medium. For melatonin treatment, the IVF zygotes were cultured in KSOM + AA medium containing melatonin until the embryos were collected at different stages.

### Embryo Collection

Embryos were collected according to a previously described method [25]. Briefly, IVO embryos at the 4-cell, 8-cell and morula stages were obtained from the recipient females by flushing the Fallopian tube with M2 medium (at 54–56, 68–70, and 76–78 h post-hCG, respectively), while embryos at the blastocyst stage were obtained from recipient females by flushing the uterus with M2 medium (96−100 h post-hCG).

The IVP embryos were collected at different stages on the basis of their developmental progress and embryo morphology, as described in previous reports [3, 4, 26].

### High-throughput RNA Sequencing

Total RNAs were extracted from blastocysts using the TRIzol reagent (Invitrogen, Carlsbad, CA, USA). Polyadenylated RNAs were isolated using an Oligotex mRNA midi kit (Qiagen, Valencia, CA, USA). RNA-seq libraries were constructed using a SOLiD whole transcriptome analysis kit (Applied Biosystems, Foster City, CA, USA) according to the standard protocol, and sequenced on an Applied Biosystems SOLiD platform to generate high-quality single-end reads. The raw reads were aligned to genome sequences, after trimming off a nucleotide each from the 5ʹ- and 3ʹ-ends and allowing up to two mismatches. Reads mapped to multiple locations were discarded and only uniquely mapped reads were used for subsequent analysis. The gene expression levels were measured as reads per kilobase of exon model per million mapped reads (RPKM). Biological processes in response to different physiological conditions were annotated using the database for annotation, visualization, and integrated discovery (DAVID v6.7; http://david.abcc.ncifcrf.gov). Differentially expressed proteins (DEPs) were run through the Search Tool for the Retrieval of Interacting Genes/Proteins (STRING; http://string.embl.de/) to build a protein-protein interaction (PPI) network, and for comparison. Phenotype annotations of the DEPs were analyzed using the Mouse Genome Informatics database (MGI; http://www.informatics.jax.org/phenotypes.shtml).

### Immunofluorescence and Immunofluorescence Quantification

To detect the abundance and distribution of actin filaments, fluorescein isothiocyanate labeled phalloidin (FITC- phalloidin) was used. FITC- phalloidin was prepared in accordance with the manufacturer's instructions. Briefly, FITC- phalloidin was dissolved as a stock solution (0.1 mg/ml) in DMSO and stored at −20°C. The stock solution was later diluted to the working concentrations (5 μg/ml) in PBST-PVA (0.2% Triton-X100 and 0.1% polyvinyl alcohol [PVA] in PBS) before use. Embryos were fixed with 3.7% formaldehyde for 1 h at 4°C, and then permeabilized in PBST-PVA for 20 min at room temperature. After washing three times with 0.1% PBS/PVA at 37°C for 5 min, the embryos were incubated with FITC- phalloidin overnight at 4°C. After washing three times with 0.1% PBS/PVA at 37°C for 5 min, the embryos were counterstained with 4’,-6-diamidino-2-phenylindole (DAPI; Vector Laboratories, Burlingame, CA, USA) for 10 min and mounted on glass-bottomed culture dishes (Nest) with Vectashield mounting medium (Vector Laboratories, Burlingame, CA, USA).

Fluorescence signals were observed using a confocal scanning laser microscope (Digital Eclipse C1, Nikon, Japan). As described previously [27], we generated a Z-stack using the acquisition function: choosing the upper and lower limits of the Z-stack, and then Z-stack images with 14 consequential sections for each embryo were taken. The full project image was generated from the Z-stack files. For all embryos identical image acquisition (laser power, light path, objective, gains and offsets) and Z-stack settings were used to minimize potential differences in signal strength from embryo to embryo. Moreover, to minimize bleaching, the embryos were not exposed to light emitted from mercury lamps and were only briefly exposed to laser light to set the Z-positions and during image acquisition.

Quantitative analysis of F-actin was carried out as described previously [28], with minor modifications. Fluorescence intensity was assessed using Image J software (NIH). Within the region of interest (ROI) in the immunofluorescence images, the average fluorescence intensity per unit area was analyzed. Three independent quantifications were performed on each embryo, and average values of all measurements were used to determine the final average intensities.

### Real-time Quantitative PCR (qPCR) Analysis

qPCR was carried out as described previously [17]. Total RNA was extracted using TRIzol reagent (Invitrogen) according to the manufacturer’s instructions. Briefly, embryos were extracted with TRIzol reagent by centrifugation, washed with 1 mL of 75% ethanol, and dissolved in 20 μL of RNase-free water. For reverse transcription, a RevertAid First Strand cDNA Synthesis Kit (Fermentas, Hanover, MD, USA) was used to generate cDNA, according to the manufacturer’s protocol. The real-time PCR amplification mixture consisted of 1 μL of cDNA, 5 μL of SsoFast EvaGreen Supermix (Bio-Rad), and 0.5 μL of both forward and reverse primers (Table 1), in a total volume of 10 μL. The PCR procedure was performed according to the manufacturer’s protocol. The PCR parameters were as follows: incubation at 95°C for 30 s; followed by 45 cycles of amplification at 95°C for 5 s, at gene-specific annealing temperature (Table 1) for 5 s, and a melting curve program at 65–95°C (starting fluorescence acquisition at 65°C, with measurements obtained at 5-s intervals until the temperature reached 95°C). Three replicates were performed, and the mRNA level of each group was normalized to the *Gapdh* mRNA level.

**Table 1:** Primer sequences used for real-time PCR.

Gene	Primer sequence (*5'-3'*)	*Tm*(°C)	Product size (bp)
*Arhgef2*	**F-**GCTGCTGATGACAGATGT; **R-**AGGCGGTCCAGAACTAAT	60	160
*β-actin*	**F-**AGGTCATCACTATTGGCAAC; **R-**ACTCATCGTACTCCTGCTTG	60	357
*Bcl2*	**F-**GATGGTGTGGTTGCCTTA; **R-**GGTATATCCGCTACAAGTTAC	60	232
*Ccdc88a*	**F-**TCCAGACACTAATGCTACAG; **R-**ATCCAGTTGCCTCTCCTT	60	196
*Coro2b*	**F-**GAGCAGACAGGCAGAATC; **R-**GAGCAAGAGGCGATGATG	60	110
*Elmo1*	**F-**GCCAACTCATTCCTCATCT; **R-**TCTCCTGTCTCCTCTCATC	60	108
*Enah*	**F-**GCTGTGATGGTCTATGATGA; **R-**AATGTGTTGTTGCCTGTATG	60	104
*Flnc*	**F-**AATTGTCACCAAGGATGCT; **R-**CACGATGATGCTGTAGTCT	60	148
*Gapdh*	**F-**TGCCCCCATGTTTGTGATG; **R-**TGTGGTCATGAGCCCTTCC	60	151
*Palld*	**F-**TTCAGGAGCGATTCTTCAG; **R-**GGACCAGCATCTTGTGAG	60	174
*Tns4*	**F-**CGAGAGCAAGCAATCAATC; **R-**CAGGAAGTGACGGATAAGG	60	171
*Vil1*	**F-**GGTGGTTAGAGAAGTTGCTA; **R-**TGGAAGAGTTGTTGGAAGAT	60	246

### Statistical Analysis

All data are presented as the means ± standard deviation (SD). One-way analysis of variance was used to compare differences among groups, followed by post-hoc comparisons between groups, using IBM SPSS statistics software version 20.

## Results

### Actin Disorganization Contributes to the Impaired Development of IVP Embryos

**Fig 1:**
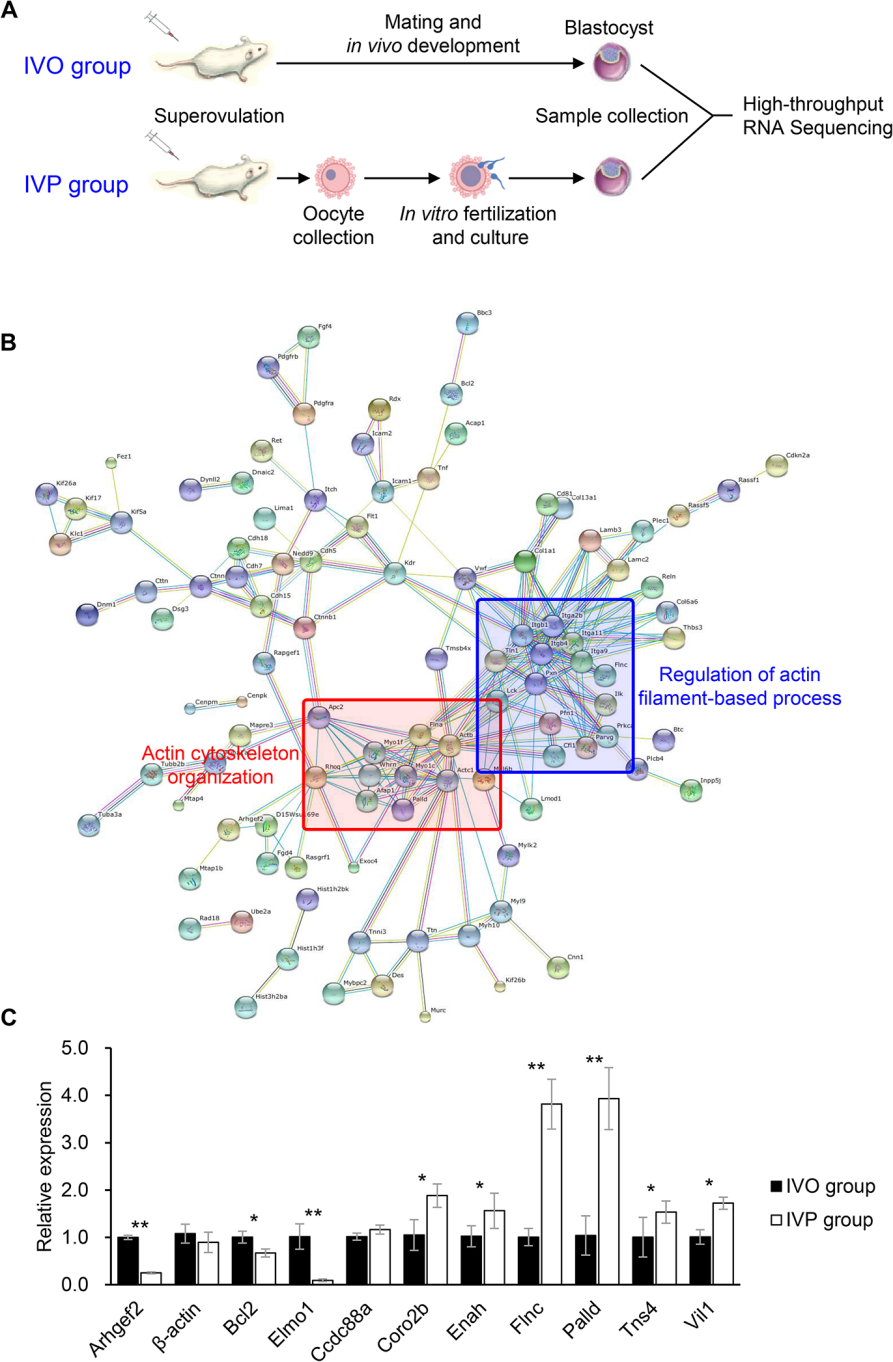
*In vitro*-produced (IVP) blastocysts are characterized by abnormal actin cytoskeleton organization. (A) Overview of the experimental design. Blastocysts were collected after either mating and *in vivo* development (IVO group) or *in vitro* fertilization and culture (IVP group). (B) Protein-protein interaction (PPI) network. Differentially expressed genes (DEGs) in the IVO and IVP blastocysts showed a tightly interconnected network, as observed from a web-based search of the STRING database. (C) Expression of actin-associated genes in the IVP and IVO blastocysts detected by quantitative real-time PCR analysis. The asterisks (* or **) in each column represent statistically significant differences (P < 0.05 and P < 0.01, respectively).

To investigate the potential effects of the IVP process on the global gene expression patterns in preimplantation embryos, we sampled 8,400 blastocysts for high-throughput mRNA sequencing (Fig 1A). The results of gene ontology (GO) classification showed that many differentially expressed genes (DEGs) were functionally associated with the “actin binding” and “cytoskeletal protein binding” clusters (Table 2). Using the DEGs that were functionally associated with cytoskeletal organization as seed nodes, a detailed PPI network was constructed (Fig 1B). Many genes that encoded actin-binding proteins or were responsible for actin organization were clustered tightly in the network. Clusters involved in actin cytoskeleton organization and the regulation of actin filament-based processes were located at the center of the network. These results indicated that cytoskeletal organization, especially actin organization, was disrupted in IVP blastocysts. We verified this observation by evaluating the expressions of some of the DEGs involved in actin organization using qPCR (Fig 1C).

**Table 2:** Representative gene ontology (GO) terms associated with actin cytoskeleton organization based on the differentially expressed genes (DEGs, fold changes > 2) between IVP and IVO blastocysts.

**Annotation Cluster 1**	**Enrichment Score: 4.54**	**Count**	**P_Value**	**Benjamini**
GOTERM_CC_FAT	extracellular region	203	0.000091	0.021
**Annotation Cluster 2**	**Enrichment Score: 4.38**	**Count**	**P_Value**	**Benjamini**
GOTERM_BP_FAT	chemical homeostasis	65	3.8E-07	0.0012
GOTERM_BP_FAT	ion homeostasis	54	0.0000014	0.0023
GOTERM_BP_FAT	homeostatic process	89	0.0000026	0.0028
GOTERM_BP_FAT	cation homeostasis	38	0.0000046	0.0036
GOTERM_BP_FAT	cellular chemical homeostasis	49	0.0000058	0.0037
GOTERM_BP_FAT	cellular ion homeostasis	48	0.0000063	0.0033
GOTERM_BP_FAT	cellular cation homeostasis	32	0.00002	0.0078
GOTERM_BP_FAT	cellular homeostasis	56	0.000036	0.013
GOTERM_BP_FAT	di-, tri-valent inorganic cation homeostasis	30	0.00006	0.017
GOTERM_BP_FAT	cellular di-, tri-valent inorganic cation homeostasis	28	0.000083	0.022
GOTERM_BP_FAT	metal ion homeostasis	23	0.00023	0.039
GOTERM_BP_FAT	cellular metal ion homeostasis	22	0.00027	0.04
GOTERM_BP_FAT	calcium ion homeostasis	20	0.00098	0.11
GOTERM_BP_FAT	cellular calcium ion homeostasis	19	0.0015	0.13
GOTERM_BP_FAT	regulation of membrane potential	19	0.025	0.5
**Annotation Cluster 3**	**Enrichment Score: 3.5**	**Count**	**P_Value**	**Benjamini**
GOTERM_CC_FAT	sarcomere	20	0.00012	0.019
GOTERM_CC_FAT	Z disc	14	0.00023	0.021
GOTERM_CC_FAT	I band	15	0.00029	0.019
GOTERM_CC_FAT	contractile fiber part	20	0.00034	0.02
GOTERM_CC_FAT	contractile fiber	21	0.00047	0.022
GOTERM_CC_FAT	myofibril	20	0.00072	0.027
**Annotation Cluster 4**	**Enrichment Score: 3.39**	**Count**	**P_Value**	**Benjamini**
GOTERM_MF_FAT	actin binding	47	0.00043	0.087
GOTERM_MF_FAT	cytoskeletal protein binding	62	0.00052	0.087
**Annotation Cluster 5**	**Enrichment Score: 2.75**	**Count**	**P_Value**	**Benjamini**
GOTERM_CC_FAT	extracellular matrix	48	0.00068	0.028
GOTERM_CC_FAT	proteinaceous extracellular matrix	46	0.00095	0.034
GOTERM_CC_FAT	extracellular region part	95	0.0058	0.14
**Annotation Cluster 6**	**Enrichment Score: 2.72**	**Count**	**P_Value**	**Benjamini**
GOTERM_MF_FAT	ion binding	456	0.0000011	0.00056
GOTERM_MF_FAT	cation binding	448	0.0000027	0.00095
GOTERM_MF_FAT	metal ion binding	442	0.0000056	0.0015
GOTERM_MF_FAT	transition metal ion binding	275	0.058	0.72
GOTERM_MF_FAT	zinc ion binding	222	0.085	0.78

**Fig 2:**
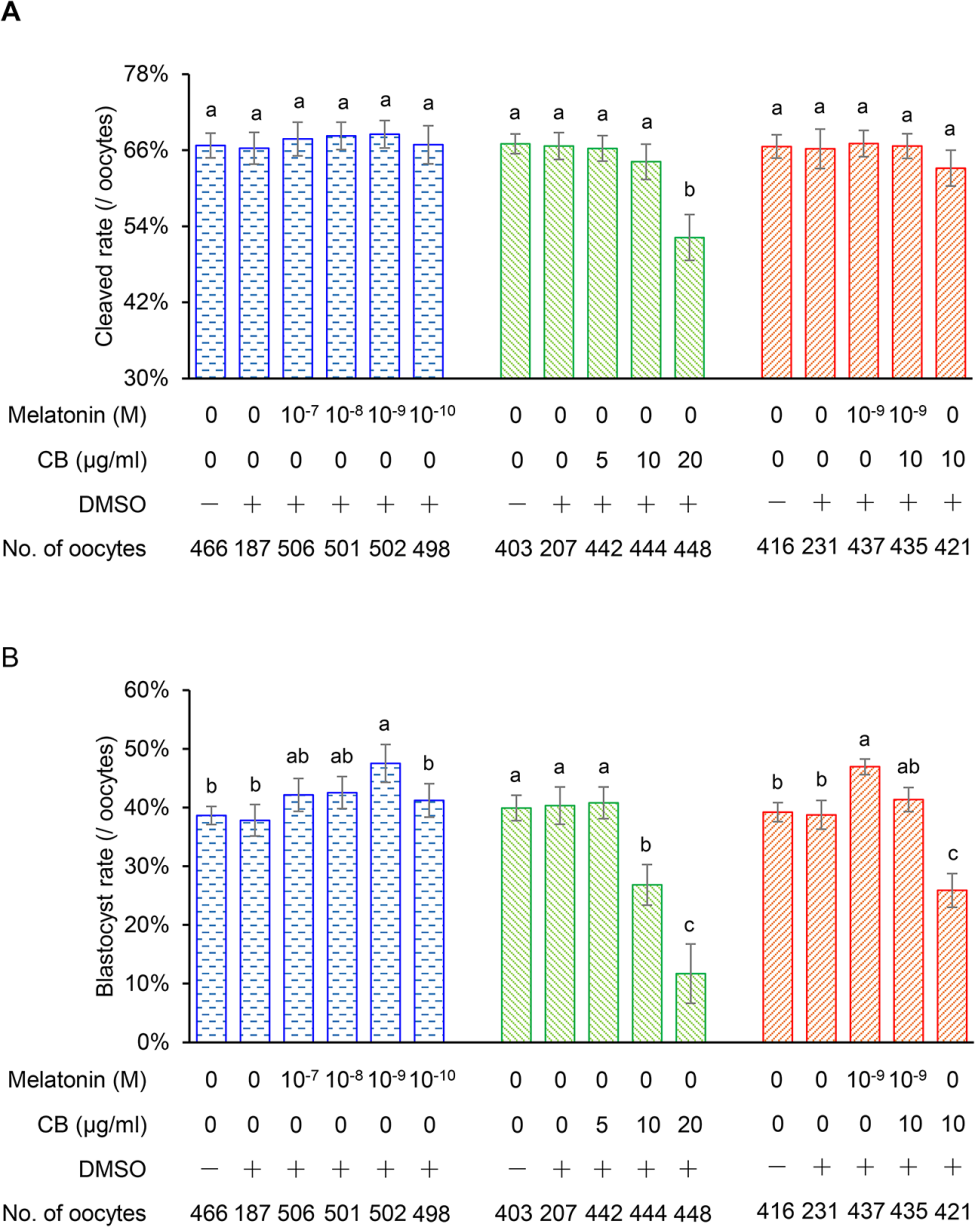
Effects of different concentrations of melatonin and/or CB supplementation on the embryonic development. (A) Cleavage rate. (B) Blastocyst rate. The vehicle control was also tested (0.1% *v/v*, the highest concentration of DMSO supplementation). All experiments were replicated at least three times independently. Different superscript letters (a, b, c) indicate significant differences (P < 0.05).

To confirm the critical role of actin organization in early embryonic development, we supplemented the culture medium with CB, which inhibits actin filament polymerization and specifically disrupts actin organization [24]. As shown in Fig 2, 10 μg/mL of CB for 3 h mildly reduced the blastocyst rate to a relatively low level, while higher concentrations (20 μg/mL) of CB led to a severe decrease in the blastocyst rate. This implied that well-organized actin is essential for *in vitro* embryonic development.

### Melatonin Improves *in vitro* Embryonic Development

As the optimal melatonin dose for *in vitro* development depends on the culture system [29] and species [15], we preselected the optimal concentration of melatonin treatment to suit the experimental conditions used in our study. There were no differences in the cleavage rates between the treatment and control groups (P > 0.05; Fig 2A). However, the blastocyst rate was significantly higher in the group treated with 10^−9^ M of melatonin than that in the control group (47.53 ± 3.22% vs. 38.67 ± 1.54%, P < 0.05) (Fig 2B). Therefore, 10^−9^ M was selected as the optimal concentration of melatonin to test the effect of melatonin treatment on the actin organization in IVP preimplantation embryos.

### Melatonin Modulates the Impaired actin Organization of IVP Embryos

**Fig 3:**
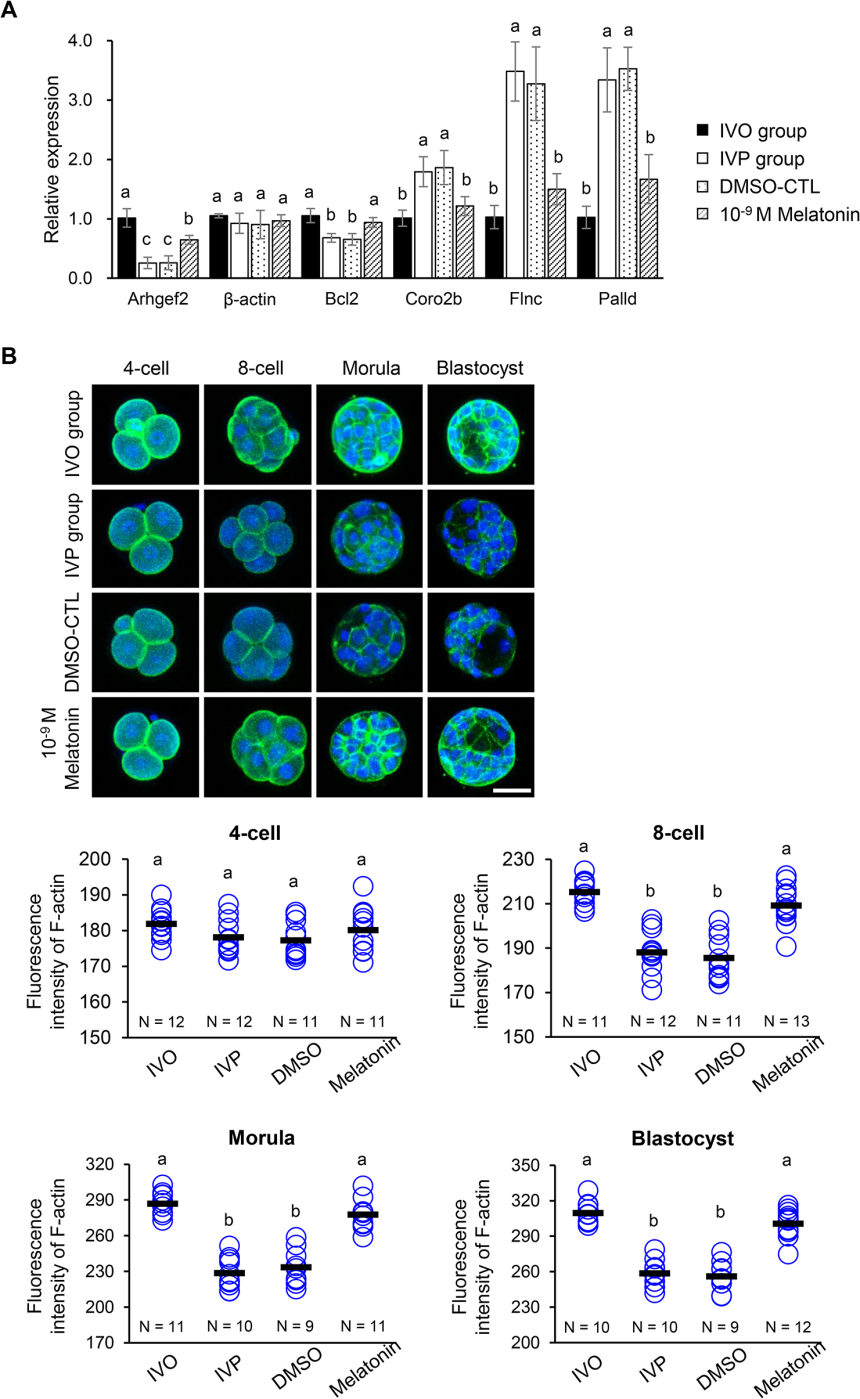
Effect of melatonin on the distribution and organization of F-actin in preimplantation embryos. (A) Comparative expression of the genes involved in actin organization in the IVP, IVO, and melatonin-treated blastocysts detected by qPCR analysis. DMSO-CTL indicates the vehicle control, in which the concentration of DMSO was 0.1% (*v/v*). (B) FITC-phalloidin immunofluorescence analysis to detect F-actin (green) in IVO, IVP, DMSO-CTL and 10^-9^M melatonin treated embryos at different stages during preimplantation development (4-cell, 8-cell, morula, and blastocyst). Nuclei (blue) are stained with 4′,6-diamidino-2-phenylindole (DAPI). Bar, 50 μm. Each circle represents the fluorescence intensity in each embryo. Horizontal lines represent the mean values. The number of embryos in each group is indicated. Different superscript letters (a, b, c) indicate significant differences (P < 0.05).

Melatonin treatment was found to alleviate or significantly reverse the disturbed expression patterns of certain genes, including *Arhgef2*, *Bcl2*, *Coro2b*, *Flnc*, and *Palld* (P < 0.05; Fig 3A). Among these genes, the expression levels of *Arhgef2* and *Bcl2* were significantly elevated (by 2.5-fold and 1.4-fold) relative to the corresponding expression levels in the control group, while the expression levels of *Coro2b*, *Flnc*, and *Palld* were significantly decreased (by 1.5-, 2.3-, and 2.0-fold, respectively) compared to the corresponding values in the control group (IVP group).

The differential expression patterns of the genes involved in actin organization led us to hypothesize that IVP embryos have faulty actin organization. Thus, we used FITC-phalloidin immunofluorescence analysis to study the distribution of F-actin, filamentous actin [30], in preimplantation IVO and IVP embryos at different stages (4-cell, 8-cell, morula, and blastocyst stages; Fig 3B). The results showed that IVP embryos had a lower abundance of F-actin (measured by fluorescence intensity of FITC-phalloidin staining) from the 8-cell stage onwards, and some cells in a proportion of the IVP morulae and blastocyst had disrupted F-actin organization. There was no obvious difference in distribution between the IVP and IVO embryos at the 4-cell stage. We quantified the average fluorescence intensities of the pixels, using previously described method [28]. The fluorescence intensity of F-actin significantly decreased in the IVP embryos from the 8-cell stage onwards compared with IVO embryos (P < 0.05). Furthermore, melatonin treatment reversed the adverse effect of the IVP process on F-actin organization (indicated by the fluorescence distribution and intensity) from the 8-cell stage onwards (average fluorescence intensity in melatonin-treated IVP embryos *vs*. control IVP embryos: 8-cell, 209.29 *vs*. 188.14; morula, 277.79 *vs*. 228.55; blastocyst, 300.66 *vs*. 258.72; P < 0.05).

### Melatonin rescues preimplantation development of the impaired IVP embryos via improved distribution and organization of F-actin

**Fig 4:**
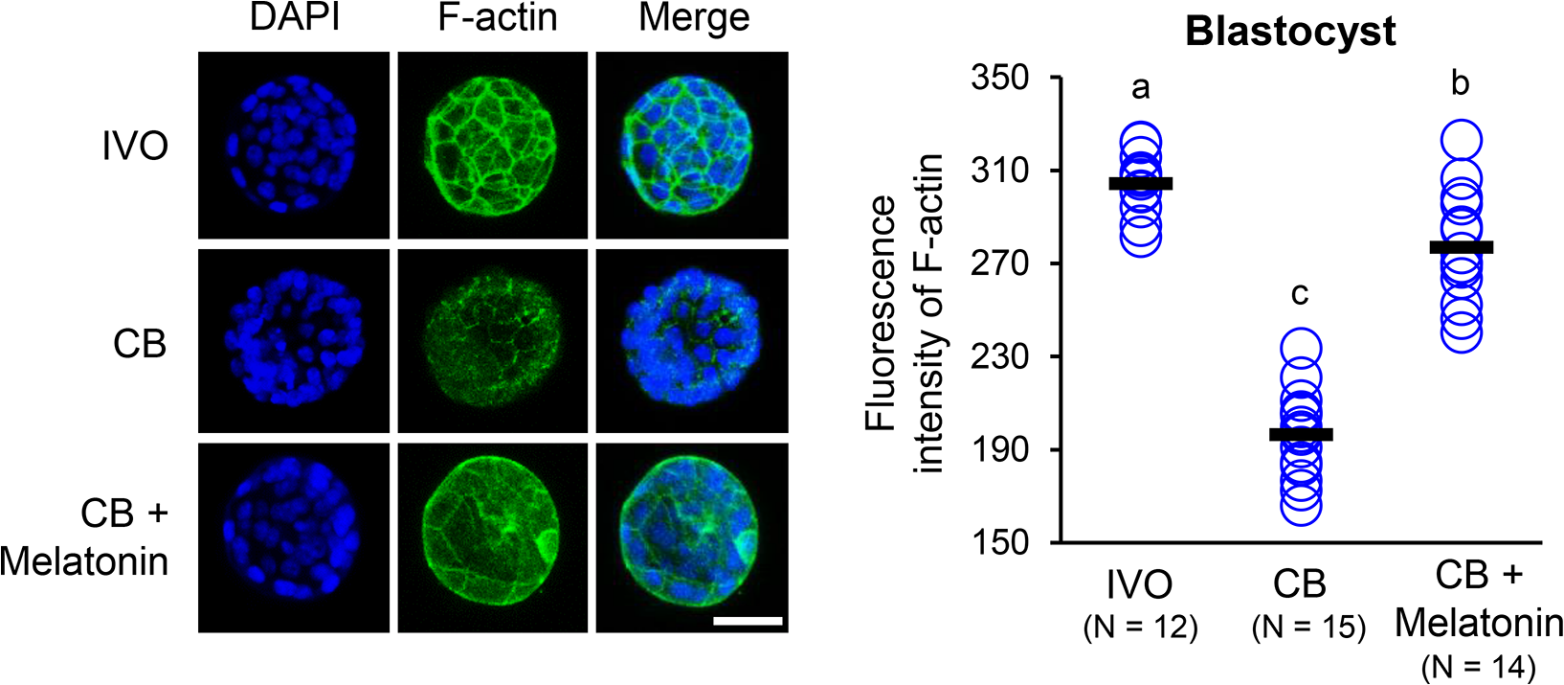
Effect of CB and/or melatonin on the distribution and organization of F-actin. Immunofluorescence analysis to detect F-actin (green) in the blastocysts of CB (10 μg/mL) and/or melatonin-treated (10^−9^ M) groups. Nuclei (blue) are stained with DAPI. Bar, 50 μm. Each circle represents the fluorescence intensity in each embryo. Horizontal lines represent the mean values. The number of embryos in each group is indicated. Different superscript letters (a, b, c) indicate significant differences (P < 0.05).

To prove that the melatonin-induced improvement of *in vitro* embryonic development at least partly depends on its actin organization-modulating role, we supplemented the culture medium with CB and melatonin. As shown in Fig 2, supplementation with 10 μg/mL of CB for 3 h mildly decreased the blastocyst rate. However, 10^−9^ M melatonin in the medium significantly alleviated the impaired embryonic development (Fig 2). Similarly, melatonin could overcome the adverse effects of treatment with 10 μg/mL CB on the distribution and organization of F-actin (Fig 4).

## Discussion

Our high-throughput RNA sequencing data suggested that an aberrant actin cytoskeleton organization might be a major factor contributing to the observed compromised *in vitro* embryonic development. Several previous studies have shown that IVP embryos undergo aberrant cytoskeletal organization. Long *et al*. showed that porcine IVP embryos were bundled or clumped, and had unevenly shaped cells [31]. Tremoleda *et al*. observed irregularly distributed microfilaments in horse IVP embryos [32]. Wang *et al*. also suggested that abnormal actin filament distribution might be a possible reason for the abnormal preimplantation development in IVP porcine embryos [33]. Functional analysis of DEGs using DAVID and STRING protein-PPI network clustering both revealed that many DEGs in the “actin cytoskeleton organization” and “regulation of actin filament-based processes” clusters were enriched. Furthermore, MGI analysis indicated that deficiency or dysregulation of these genes might lead to impaired embryonic development and mortality. For example, *Bcl2* enhances actin polymerization [34], homozygous *Bcl2*-null mutants show oocyte maturation arrest and apoptosis induction during early embryonic development [35]. *Palld* is involved in modulating the actin cytoskeleton and in nervous system development [36], and its dysregulation leads to ventral closure defects and complete lethality throughout fetal growth and development [37]. *Enah* encodes an actin regulatory protein involved in the control of cell motility and adhesion. Deficiency of this gene has been shown to cause defects in the major axonal projection pathways of the brain [38]. Taken together, these results indicated that deficiency or dysregulation of genes involved in actin organization might be a major factor leading to impaired early embryonic development.

Our immunofluorescence analysis focused on the aberrant actin organization in IVP embryos. We found that F-actin was diffusely distributed in IVP embryos from the 8-cell stage onwards. Actin is a multifunctional protein that participates in many important cellular processes, such as cell division and cytokinesis, cell signaling, and the establishment and maintenance of cell junctions and cell shape [39–41]. Many of these processes are mediated by extensive and intimate interactions of actin with cellular membranes [42]. This diffuse distribution pattern of F-actin indicated that actin might not function properly in IVP embryos. Furthermore, quantification of the fluorescence intensity showed a significantly reduced abundance of F-actin in IVP embryos compared with IVO embryos. Studies have shown that deficiency or dysregulation of F-actin leads to a series of abnormal phenotypes, such as retarded embryogenesis, decreased body growth/size, and mortality/aging [43, 44]. Actin plays a crucial role in various dynamic events during oogenesis, fertilization, and embryonic development [45]; therefore, the decreased abundance as well as the suboptimal distribution, might adversely affect *in vitro* embryonic development. The detrimental effect of actin organization on early *in vitro* development was further confirmed by CB-induced actin disorganization, which dramatically decreased the developmental rate of IVP embryos.

Previous studies indicated that melatonin could improve the efficiency and quality of *in vitro* preimplantation development [15] and could increase the implantation rate after embryo transfer [46]. These beneficial effects of melatonin are frequently associated with its free radical scavenging and antiapoptotic abilities [19]. Considering the function of melatonin in actin organization, we questioned whether the beneficial effect of melatonin on *in vitro* embryonic development is dependent on the improved actin organization.

Our data demonstrated that melatonin could modulate actin organization in preimplantation embryos, which in turn might improve *in vitro* embryonic development. The results of the immunofluorescence analysis revealed that melatonin treatment improved the organization and distribution of F-actin, which mirrored the increased blastocyst rate. Moreover, the melatonin-mediated improvement of F-actin organization could be explained by the reversion to normal expression levels of dysregulated genes involved in actin organization (*Arhgef2*, *Bcl2*, *Coro2b*, *Flnc*, and *Palld*). It should also be mentioned that the effects of melatonin on actin organization are not mediated by membrane receptors. In a previous study, melatonin-induced microfilament rearrangement and microtubule enlargement was found to occur even in the presence of large concentrations of the MT1 and MT2 melatonin membrane receptor antagonist luzindole [47]. In addition, considering the fact that preimplantation embryos do not express these two melatonin membrane receptors [17, 48], we hypothesized that melatonin’s involvement in the modulation of actin organization is receptor independent.

To further confirm that actin disorganization is an essential factor that impairs the development of IVP embryos, we tested whether melatonin could alleviate CB-induced actin disorganization and rescue the impaired embryonic development. CB is a well-known specific inhibitor of actin filament polymerization that reduces the actin polymerization rate and inhibits the interaction of actin filaments in solution; however, CB has little effect on the rate of monomer addition and the rate of filament annealing [49]. When the *in vitro* culture medium was supplemented with CB alone, the blastocyst rate of IVP embryos significantly decreased, and the distribution of F-actin was severely disrupted. In contrast, the aberrant actin organization in IVP embryos after combined treatment with CB and melatonin appeared to be rescued, with a developmental rate comparable to IVP embryos. These results suggested that melatonin could restore the impaired embryonic development caused by actin disorganization, probably by improving actin organization.

## Conclusions

Our study used high-throughput data, immunofluorescence and *in vitro* experiments using mouse embryos cultured with CB to study the effect of actin organization on IVP preimplantation embryos. Our results revealed that aberrant actin organization might contribute considerably to the impaired development of IVP preimplantation embryos. Furthermore, melatonin treatment improved actin organization and showed an ability to rescue this impaired development. Our results demonstrated that actin disorganization would be a reasonable target to optimize existing IVP systems. This study identified a promising strategy to improve the clinical ART process.
